# Amphiphilic dendrons as supramolecular holdase chaperones†

**DOI:** 10.1039/d3cb00086a

**Published:** 2023-09-08

**Authors:** Elizabeth R. Piedmont, Erin E. Christensen, Todd D. Krauss, Benjamin E. Partridge

**Affiliations:** a Department of Chemistry, University of Rochester Rochester NY 14627-0216 USA benjamin.partridge@rochester.edu; b Institute of Optics, University of Rochester Rochester NY 14627-0186 USA

## Abstract

The aggregation of incompletely or incorrectly folded proteins is implicated in diseases including Alzheimer's, cataracts, and other maladies. Natural systems express protein chaperones to prevent or even reverse harmful protein aggregation. Synthetic chaperone-like systems have sought to mimic the action of their biological counterparts but typically require substantial optimization and high concentrations to be functional, or lack programmability that would enable the targeting of specific protein substrates. Here we report a series of amphiphilic dendrons that undergo assembly and inhibit the aggregation of fragment 16–22 amyloid β protein (Aβ_16–22_). We show that monodisperse dendrons with hydrophilic tetraethylene glycol chains and a hydrophobic core based on naphthyl and benzyl ethers undergo supramolecular assembly in aqueous solutions to form sphere-like particles. The solubility of these dendrons and their assemblies is tuned by varying the relative sizes of their hydrophilic and hydrophobic regions. Two water-soluble dendrons are discovered and shown, *via* fluorescence experiments with rhodamine 6G, to generate a hydrophobic environment. Furthermore, we demonstrate that sub-stoichiometric concentrations of these amphiphilic dendrons stabilize Aβ_16–22_ peptide with respect to aggregation, mimicking the activity of holdase chaperones. Our results highlight the potential of these amphiphilic molecules as the basis for a novel approach to artificial chaperones that may address many of the challenges associated with existing synthetic chaperone mimics.

## Abstract 2

Supramolecular capsules assembled from amphiphilic dendrons based on naphthyl and benzyl ethers exhibit chaperone-like activity by reducing the extent of fibrillation of an amyloid beta protein fragment.

## Graphical abstract

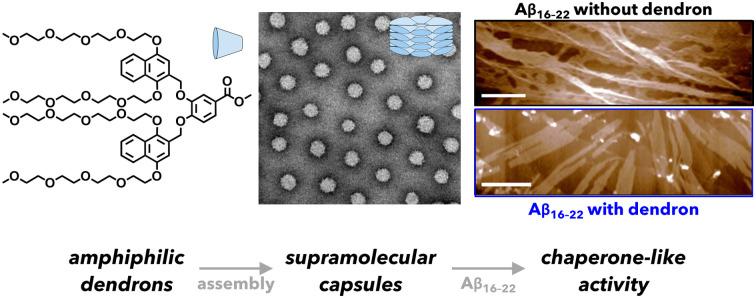

## Introduction

Proteins are important biomolecules that are involved in most biological processes in living systems. Their structure is typically integral to their function; therefore, to ensure that proteins adopt the appropriate secondary and tertiary structures, protein folding is often mediated by molecular chaperones.^1,2^ When the integrity of this process is lost, misfolded or unfolded proteins can aggregate due to exposed hydrophobic residues.^3^ Aggregation is implicated in diseases such as Alzheimer's, Parkinson's, type 2 diabetes, and Huntington's disease. To address this issue, artificial systems with chaperone-like functions mimicking those found in Nature have been explored.^4^ Two main classes of artificial chaperones have emerged: those that function based on the hydrophobic effect and those based on electrostatic^5–7^ interactions. Hydrophobic systems can be categorized as low molecular weight (MW) (*e.g.*, detergents,^8,9^ cyclodextrins^10,11^) and high MW (*e.g.*, poly(ethylene glycol),^12,13^ poly(*N*-isopropylacrylamide)^14,15^). These chaperones have been shown to successfully refold proteins and prevent aggregation. However, low MW (molecular) systems require substantial optimization for each protein because small changes to their molecular structure lead to drastic changes in chaperone behavior, thereby limiting peptide substrate scope.^16^ In contrast, high MW (polymeric) systems exhibit broad substrate scope but cannot easily target specific proteins due to their polydispersity. Moreover, many systems interact too strongly with their protein substrates, hindering purification and protein release, lowering refolding yields, and limiting their application as therapeutics.^4,17^ Therefore, an unsolved challenge in this field is the development of adaptive, broadly applicable systems that permit tuning of the interactions between the protein and the chaperone.

**Fig. 1:**
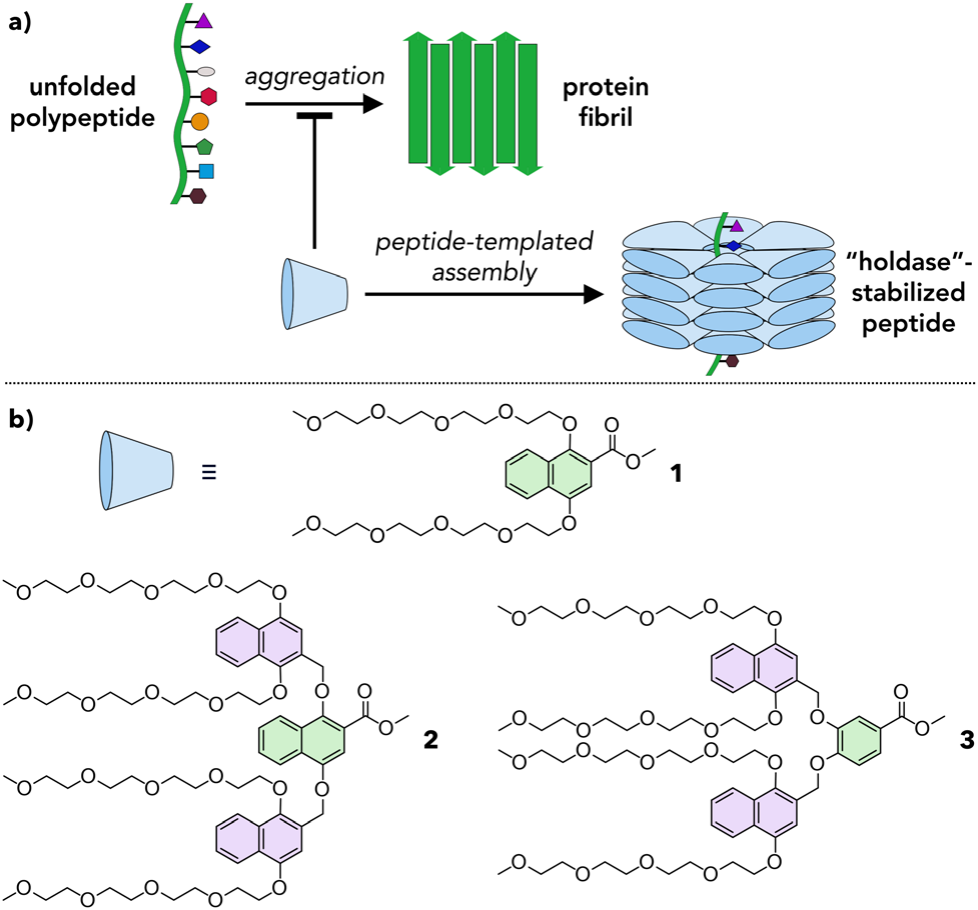
Supramolecular chaperones. (a) Schematic of a proposed design for a supramolecular chaperone inhibiting peptide aggregation. Blue cone represents an individual monomer. (b) Molecular structures of amphiphilic dendrons 1, 2, and 3 explored as monomers in this work. Green and purple aromatic rings highlight first and second generation branching points, respectively.

Herein we propose a new approach based on amphiphilic dendrons that assemble into dynamic, adaptive capsules that interact with proteins to inhibit their aggregation (Fig. 1). Dendrons are branched molecules whose structure is built up through iterative generations, providing access to macromolecular scaffolds with molecular precision.^18^ The dendrons reported here are of a sufficient MW (up to ∼1300 g mol^−1^) to permit fine tuning of their chemical structure while still benefitting from the monodispersity inherent in molecular systems. In this proof-of-concept study we synthesize a series of amphiphilic dendrons based on naphthyl ethers, show that these dendrons form supramolecular assemblies in a structure-dependent manner in water, and demonstrate that they reduce the aggregation of a prototypical amyloid fragment.

## Results and discussion

Natural chaperones such as the GroEL/GroES pair sequester misfolded peptides in a hydrophobic cavity to inhibit their aggregation.^19^ We hypothesized that amphiphilic dendrons would serve as effective mimics of such systems (Fig. 1a). First, their amphiphilic nature will induce spontaneous supramolecular assembly in aqueous solutions.^20^ Second, their hydrophobic functional groups permit interactions with misfolded peptides. Third, the branched structure and modular synthesis of the dendrons facilitate tuning of the relative size of the hydrophobic and hydrophilic regions.

Accordingly, first and second generation naphthyl ether dendrons 1 and 2 (Fig. 1b) were synthesized. Naphthyl ethers^21^ were chosen as the hydrophobic component due to their extended, planar aromatic system and tetraethylene glycol was chosen as the hydrophilic component due to its water solubility. The synthetic route (Scheme S1, ESI†) was based on a methodology developed by Percec and coworkers for the synthesis of benzyl ether dendrimers,^21–23^ but utilized methyl 1,4-dihydroxynaphthoate (7) as the branching unit (details in ESI†).

Whereas first generation 1 was found to be highly soluble in water at concentrations of at least 1000 μM, second generation 2 was insoluble in water and needed a polar organic solvent, such as MeCN, to dissolve fully. Even so, the solubility of 2 was limited to ∼50 μM in 10% MeCN in water (hereafter denoted 10% aq. MeCN) and ∼200 μM in 20% aq. MeCN. To maintain water solubility at higher concentration, an alternate second generation dendron, 3, was designed. The structure of 3 is identical to that of 2 except that the first generation naphthyl ether branching unit is replaced by a smaller, benzyl ether unit (compare green units in Fig. 1b). This small reduction in the degree of hydrophobic character was sufficient to confer 3 with solubility in water to concentrations of at least 250 μM.

**Fig. 2:**
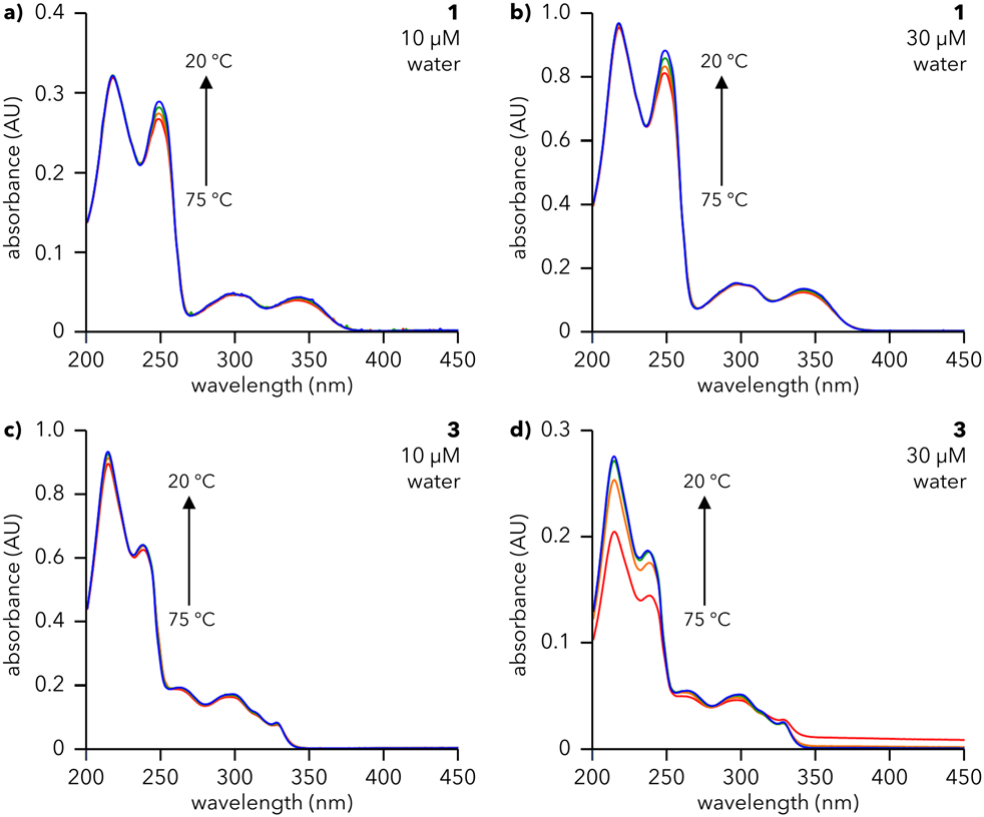
UV-vis studies of 1 and 3 in solution. UV-vis spectra of (a) and (b) 1 and (c) and (d) 3 collected upon cooling aqueous solutions at the indicated concentrations from 75 °C (red line) to 20 °C (blue line) at 0.5 °C min^−1^. Spectra were collected at 75, 60, 40, and 20 °C. Spectra in (a)–(c) were collected in a cuvette with path length (*l*) of 10 mm; spectra in (d) were collected in a cuvette with *l* = 1 mm.

The assembly of dendrons 1, 2, and 3 in solution was investigated using UV-vis spectroscopy. Solutions of 1 and 3 were prepared in water (Fig. 2) while those of 2 were prepared in 10% aq. MeCN due to its insolubility in pure water (Fig. S1, ESI†). The absorption spectra for all compounds at 20 °C (blue lines in Fig. 2 and Fig. S1, ESI†) show major peaks around 225 and 245 nm, corresponding to π → π* transitions,^24–26^ and above 300 nm, arising from *n* → π* transitions.^27^ Cooling solutions of 1, 2, and 3 from 75 to 20 °C lead to structure- and concentration-dependent spectral changes. Spectra of 3 in water exhibit a substantial increase in absorbance at 217 and 249 nm upon cooling (Fig. 2d), even for concentrations as low as 30 μM, signifying that 3 assembles under these conditions. The same trend is observed for 2 in 10% aq. MeCN (Fig. S1, ESI†). This effect becomes more pronounced as the concentrations of 2 and 3 are increased (Fig. S1 and S2, ESI†), indicative of a higher degree of assembly at higher concentrations. Furthermore, the presence of an isosbestic point in the spectra of 2 and 3 (∼255 and ∼316 nm, respectively) denotes a one-to-one transition between two species (*e.g.*, monomeric species and an assembly). In contrast, the spectra of 1 at 20 °C (blue lines, Fig. 2a and b) are almost identical to those at 75 °C (red lines, Fig. 2a and b), with a minor increase in the intensity of the feature at 249 nm. This observation is valid across a range of concentrations of 1 in water, from 10 μM (Fig. 2a) to 200 μM (Fig. S3, ESI†). Atomic force microscopy (AFM) data, to be discussed later, show that both 1 and 3 form supramolecular assemblies. We attribute the differences in their temperature-dependent UV spectra to the different arrangements of aromatic moieties in first generation 1*vs.* second generation 3. Furthermore, we note that the small changes in absorbance observed for 1 are consistent with previous reports of naphthalene-containing supramolecular structures.^28^

To assess the reversibility of the assembly process, 30 μM solutions of 1 (water), 2 (10% aq. MeCN), and 3 (water) were subjected to three cycles of heating and cooling (20 to 75 °C, 0.5 °C min^−1^) and their absorbance monitored at 217 and 249 nm (Fig. S4, ESI†). 1 and 3 show smooth, reversible changes in absorbance, characteristic of reversible assembly, while 2 exhibits increasingly large changes in absorbance with each subsequent heating and cooling cycle, suggesting that assembly is not reversible or is hysteretic. Therefore, UV-vis studies reveal that dendrons 1 and 3 undergo reversible, temperature-dependent assembly in water. Conversely, dendron 2 is insoluble in water and irreversibly assembles in 10% aq. MeCN; hence 2 was not explored further.

**Fig. 3:**
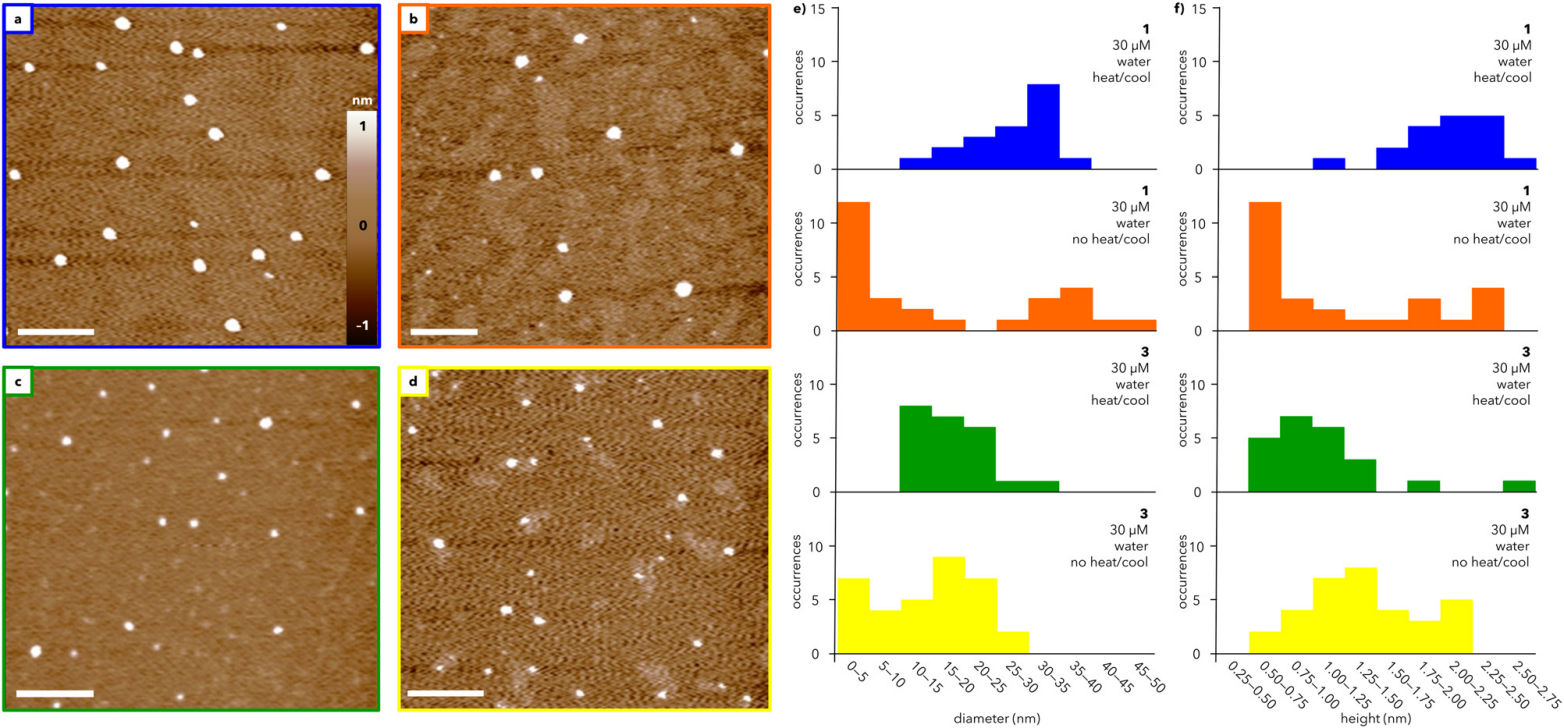
AFM studies of assemblies formed from 1 and 3. AFM height images of (a) and (b) 1 and (c) and (d) 3 spin-coated on mica substrates prepared from 30 μM solutions in water that were (a) and (c) heated to 75 °C and subsequently cooled to 25 °C (at 0.5 °C min^−1^) or (b) and (d) imaged immediately after dissolution at 25 °C. Scale bars = 200 nm. Histograms showing the (e) diameters and (f) heights of particles in (a)–(d), denoted by color.

The morphologies of the assemblies formed from water-soluble dendrons 1 and 3 were characterized using AFM (Fig. 3). Solutions of 1 and 3 were heated and slowly cooled from 75 to 25 °C at 0.5 °C min^−1^ and subsequently spin-coated on mica (Fig. 3a and c; full sample preparation details in ESI†). AFM images of heated and cooled solutions (30 μM) of 1 and 3 show the presence of round features, with similar diameters (12–35 nm and 12–24 nm, respectively; Fig. 3e) and heights (1.2–2.6 nm and 0.8–2.6 nm, respectively; Fig. 3f). Increasing the concentration of 1 and 3 to 50 μM did not substantially alter the observed structures (compare Fig. 3 and Fig. S5, ESI†). Transmission electron microscopy (TEM) measurements of 3 (Fig. S8, ESI†) support the formation of well-defined particles with diameters ranging primarily from 25–35 nm. The larger size visualized by TEM compared to AFM is consistent with previous microscopy studies on organic nanoparticles.^29^ In contrast, TEM images of 1 do not exhibit well-defined particles, potentially due to stain-induced changes in assembly, though some particle-like structures were observed (further discussion in ESI†).

Amplitude images measured by AFM show domed features suggestive of collapsed spheres rather than flat discs (Fig. S6, ESI†). Whether such spheres are hollow or solid could not be determined from these AFM data, but the aspect ratio of these features is consistent with a previous report that attributed a ∼10 : 1 diameter/height ratio visualized by AFM to soft, hollow spheres.^30^ This aspect ratio can be rationalized by analogizing the drying of a hollow supramolecular capsule on a surface to deflating a basketball, whereby the particle diameter is almost invariant but the height decreases substantially.

To investigate the role of temperature, solutions of 1 and 3 at 30 μM were directly spin-coated on mica without heating and cooling (Fig. 3b and d). Under these conditions, both 1 and 3 assemble into a broader range of particles with a marked increase in smaller features compared to the heated and cooled samples (Fig. 3e and f; compare blue and orange histograms for 1 and green and yellow histograms for 3). The range of particle sizes is larger for 1 than for 3, suggesting that heating and cooling has less of an impact on the assembly of 3 than that of 1. In both cases, heating and cooling leads to an overall improvement in the circularity of the particles, which is a measure of how closely the particles resemble a perfect circle (Fig. S7, ESI†). Notably, the range of circularity values for 1 is substantially narrower after heating and cooling than the range measured for 3. Together, the UV and AFM data show that both 1 and 3 generate spherical-like aggregates with low dispersity, though the exact mechanism of assembly warrants further study.

Having established that 1 and 3 form discrete assemblies in solution, we explored whether these assemblies could define a hydrophobic environment within an aqueous solution. Rhodamine 6G is a water-soluble fluorescent dye that has been used to monitor molecular assembly in solution due to its solvation-dependent emission.^31,32^ Specifically, the fluorescence intensity of rhodamine 6G decreases as the dye becomes less solvated by water, that is, when the dye occupies a more hydrophobic environment. Upon excitation at 488 nm, rhodamine 6G exhibits a strong emission peak centered at 550 nm (Fig. S9, black line, ESI†). Addition of 1 or 3 decreases the intensity of this emission, which reduces further with increasing concentrations of dendron (Fig. S9, ESI†). This behavior indicates that the rhodamine dye is becoming less well solvated by water as the concentration of dendron increases, suggesting that the dye is experiencing a more hydrophobic environment defined by the presence of 1 or 3.

Encouraged by fluorescence studies, we investigated how assemblies of 1 and 3 would interact with a hydrophobic peptide. Fragment 16–22 of amyloid β protein (Aβ_16–22_) was chosen as a model peptide because of its biological relevance and well-studied aggregation.^33,34^ Circular dichroism (CD) spectra of a solution of Aβ_16–22_ (200 μM in water) showed characteristic β-sheet formation that was complete within 30 min (Fig. S10a, ESI†). Compared to that of an ideal β-sheet, the CD spectrum of Aβ_16–22_ lacks a negative peak around 220 nm, which has been attributed to β-sheet twisting that reduces the extent of hydrogen bonding.^35,36^

**Fig. 4:**
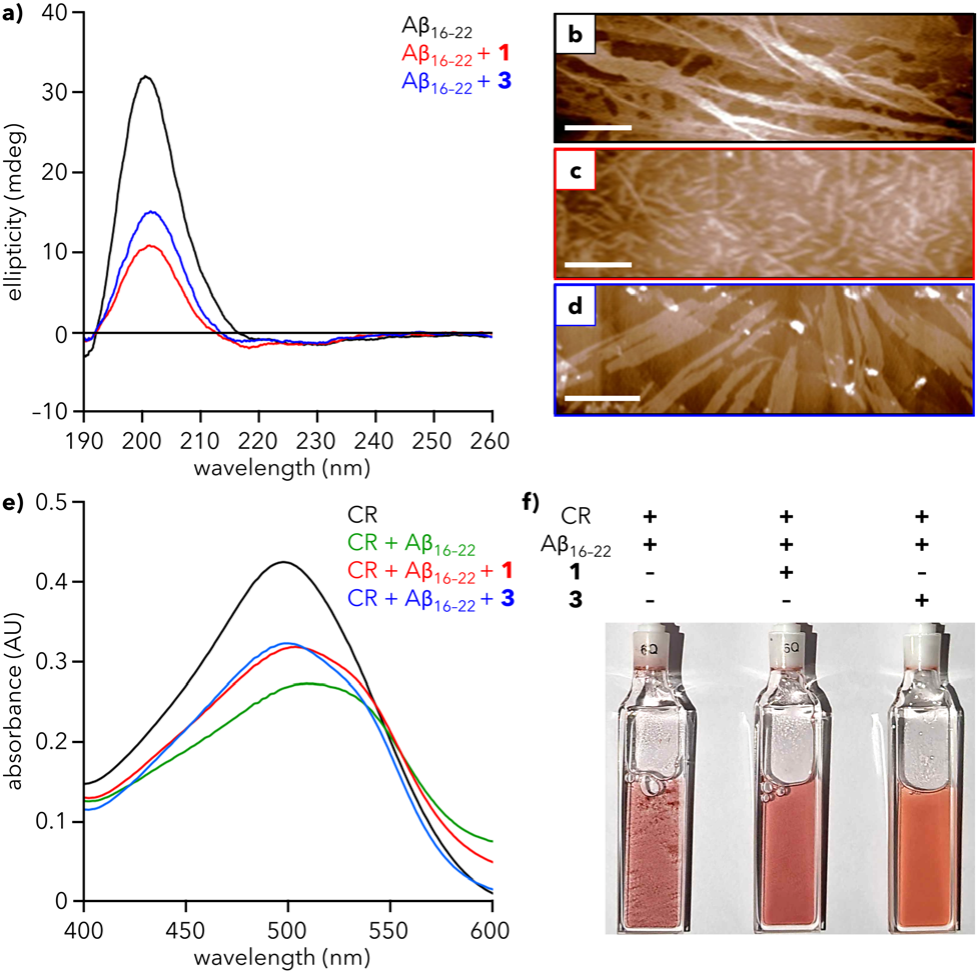
Effect of dendrons on Aβ_16–22_ fibrillation. (a) CD spectra of Aβ_16–22_ alone (200 μM) and with 1 and 3 (30 μM) in water at 25 °C. (b)–(d) AFM images of (b) Aβ_16–22_ alone and with (c) 1 and (d) 3. Scale bars = 500 nm. (e) UV-vis spectra of Congo red (CR) alone (100 μM), with Aβ_16–22_ (200 μM), and with 1 and 3 (30 μM) in water at 25 °C after 1 h. Spectra at 0 h are provided in Fig. S12 (ESI†). (f) Photograph of the samples from (e) after 26 h. Precipitate formation is most visible in the cuvettes containing CR + Aβ_16–22_.

Addition of Aβ_16–22_ to a solution of either 1 or 3 leads to a substantial decrease in the intensity of the CD signal at 200 nm (Fig. 4a), demonstrating that both dendrons reduce the extent of Aβ_16–22_ fibrillation in solution. To further support this observation, we sought to monitor fibrillation using the commonly used amyloid probe, thioflavin T (ThT). However, mixtures of ThT and dendron led to an increase in fluorescence that precluded the use of ThT in our system (Fig. S11, ESI†). Therefore, Congo red (CR) was chosen as an alternative. CR is a known colorimetric probe for assessing protein aggregation, characterized by a shift in maximum absorbance (*λ*_max_) from 490 to 540 nm upon binding to amyloid fragments.^37,38^ Addition of Aβ_16–22_ to a solution of CR leads to formation of a shoulder at ∼540 nm and overall reduction in absorbance after 1 h (Fig. 4e and Fig. S12, ESI†), suggesting that Aβ_16–22_ is aggregating and CR is binding to those aggregates. In contrast, when Aβ_16–22_ was added to solutions containing CR and either 1 or 3, the decrease in absorbance and shift in *λ*_max_ are reduced, indicative of a reduced extent of fibrillation. After ∼1 day, the mixture of Aβ_16–22_ and CR exhibited visible precipitate formation, which was substantially reduced in mixtures containing 1 or 3 (Fig. 4f and Fig. S13, ESI†).

To visualize the reduction of fibrillation, solutions of Aβ_16–22_ with 1 or 3 were analyzed using AFM. Aβ_16–22_ alone assembles into a dense array of extended fibers (Fig. 4b). In contrast, mixtures of Aβ_16–22_ and 1 or 3 show a reduction of the peptide fiber length, with the greater reduction observed for 1 (Fig. 4c), consistent with CD data (red line, Fig. 4a). Diminished fibrillation is also apparent for the mixture of 3 and Aβ_16–22_ (Fig. 4d). Analysis of AFM images of 1 and 3 reveal an average particle height of 3.6 and 4.0 nm, respectively, in the presence of peptide, compared to 1.2 and 1.4 nm, respectively, in the absence of peptide. This increase in height could be consistent with encapsulation of Aβ_16–22_ monomers within a hollow sphere of 1 or 3, analogous to natural holdase chaperones that stabilize hydrophobic monomers against aggregation.^39^ However, more studies are needed to establish the mechanistic basis for this action.

## Conclusions

In this work, we have developed a simple synthetic mimic of a holdase chaperone. A series of amphiphilic dendrons was designed to form supramolecular assemblies in aqueous solutions. We show that deliberate changes to the molecular structure of these dendrons program their solubility, leading to two molecules, 1 and 3, that assemble in pure water. Remarkably, these two molecules inhibit the fibrillation of a model peptide at low, sub-stoichiometric quantities (30 μM dendron *vs.* 200 μM peptide), which compares favorably to reported systems that often require millimolar concentrations.^10,11,13,40^ Importantly, the monodispersity and synthetic addressability of these amphiphilic constructs will enable the development of structure–property relationships that correlate molecular structure with chaperone activity. Taken together, this study establishes a new approach to the design of molecular chaperones.

## Conflicts of interest

There are no conflicts of interest to declare.

## Supplementary Material

CB-004-D3CB00086A-s001
