# Supplementary material for: Amphiphilic dendrons as supramolecular holdase chaperones
Source: RSC Chem Biol. 2023 Sep 8;4(10):754–9. doi: 10.1039/d3cb00086a (PMC10549246; doi:10.1039/d3cb00086a)
Supplement: CB-004-D3CB00086A-s001 [file CB-004-D3CB00086A-s001.pdf]

Electronic Supplementary Material (ESI) for RSC Chemical Biology.
This journal is © The Royal Society of Chemistry 2023

Supplementary Information for

Amphiphilic Dendrons as Supramolecular Holdase Chaperones

Elizabeth R. Piedmont,[1] Erin E. Christensen,[1] Todd D. Krauss,[1,2] and Benjamin E. Partridge*,[1]

[1]*Department of Chemistry, University of Rochester, Rochester, NY 14627-0216, United States.*

[2]*Institute of Optics, University of Rochester, Rochester, NY 14627-0186, United States.*

*Email: benjamin.partridge@rochester.edu

Table of Contents

1. Synthesis and Characterization of Amphiphilic Minidendrons S2
   1.1. Materials and Methods S2
   1.2. Experimental Procedures S3
2. Synthesis of Aβ_{16-22} Peptide S7
3. UV-vis Spectroscopy S7
   3.1. Materials and Methods S7
   3.2. Additional Data S8
   3.3. Reversibility Study S10
4. Atomic Force Microscopy S11
   4.1. Materials and Methods S11
   4.2. Additional Data S11
5. Transmission Electron Microscopy S13
   5.1. Materials and Methods S13
   5.2. TEM Data S13
6. Fluorescence Spectroscopy S14
   6.1. Materials and Methods S14
   6.2. Fluorescence Studies with Rhodamine 6G S14
7. Circular Dichroism Spectroscopy S15
   7.1. Materials and Methods S15
   7.2. Additional Data S15
8. Amyloid Binding Assays S16
   7.1. Thioflavin T S16
   7.2. Congo Red S16
9. References for the Supplementary Information S18

1. Synthesis and Characterization of Amphiphilic Minidendrons

1.1. Materials and Methods

Materials. Anhydrous solvents were used in all reactions except for the synthesis of **2** and were either prepared from commercial solvents using a Pure Process Technology Solvent Purification System (CH_2Cl_2 and THF) or purchased as anhydrous reagents directly from Thermo Fisher Scientific (CH_2Cl_2, THF and DMF). $NaHCO_3$ used for esterification reactions was stored in a desiccator to prevent water adsorption. LiCl and $LiAlH_4$ (2.4 M in THF) (both from Acros), $NaHCO_3$ (from EMD Millipore), HCl (12 M aq.) (from Macron Fine Chemicals), DMSO-d_6 (from Cambridge Isotope Laboratories), thionyl chloride (2 M in CH_2Cl_2) (from Sigma-Aldrich), tetraethylene glycol (**4**, from Combi-Blocks), 1,4-dihydroxy-2-naphthoic acid (**6**, from TCI Chemicals) and methyl 3,4-dihydroxybenzoate (**10**, Biosynth) were used as received. All other chemicals were purchased from Thermo Fisher Scientific and used as received. Deionized water was used for all aqueous washes and solutions.

General Methods. All reactions (except for the synthesis for **2**) were conducted under a nitrogen atmosphere using anhydrous solvents in glassware that was dried in an oven (120 °C) for at least 30 min and cooled under vacuum prior to use. Room temperature denotes ambient temperature in our laboratories, 23 ± 2 °C.

Column chromatography. Column chromatography was carried out using a Büchi Pure C-810 Flash Chromatography System using prepacked Büchi EcoFlex Silica columns as the stationary phase and the indicated solvents as mobile phase. Elution of desired products was monitored using absorption at 254 nm. Fractions were visualized by thin-layer chromatography on silica gel plates.

Nuclear magnetic resonance (NMR) spectroscopy. NMR spectra were recorded on a Bruker DPX–500 instrument at ambient temperature in DMSO-d_6. ^1H and ^{13}C spectra were referenced to the signal arising from residual non-deuterated solvent. Data are represented as follows: chemical shift, multiplicity (s = singlet, d = doublet, t = triplet, q = quartet, m = multiplet), coupling constants, integration.

Mass spectrometry. High resolution mass spectrometry (HRMS) data were recorded were recorded on a Thermo Scientific Q Exactive Plus Hybrid Quadrupole-Orbitrap mass spectrometer in electrospray ionization mode. Samples were prepared at a concentration of 10 µg/mL in a 50:50 mixture of MeCN and water + 0.1 % formic acid.

1.2. Experimental Procedures

Scheme S1. Synthesis of Amphiphilic Dendrons 1, 2, and 3

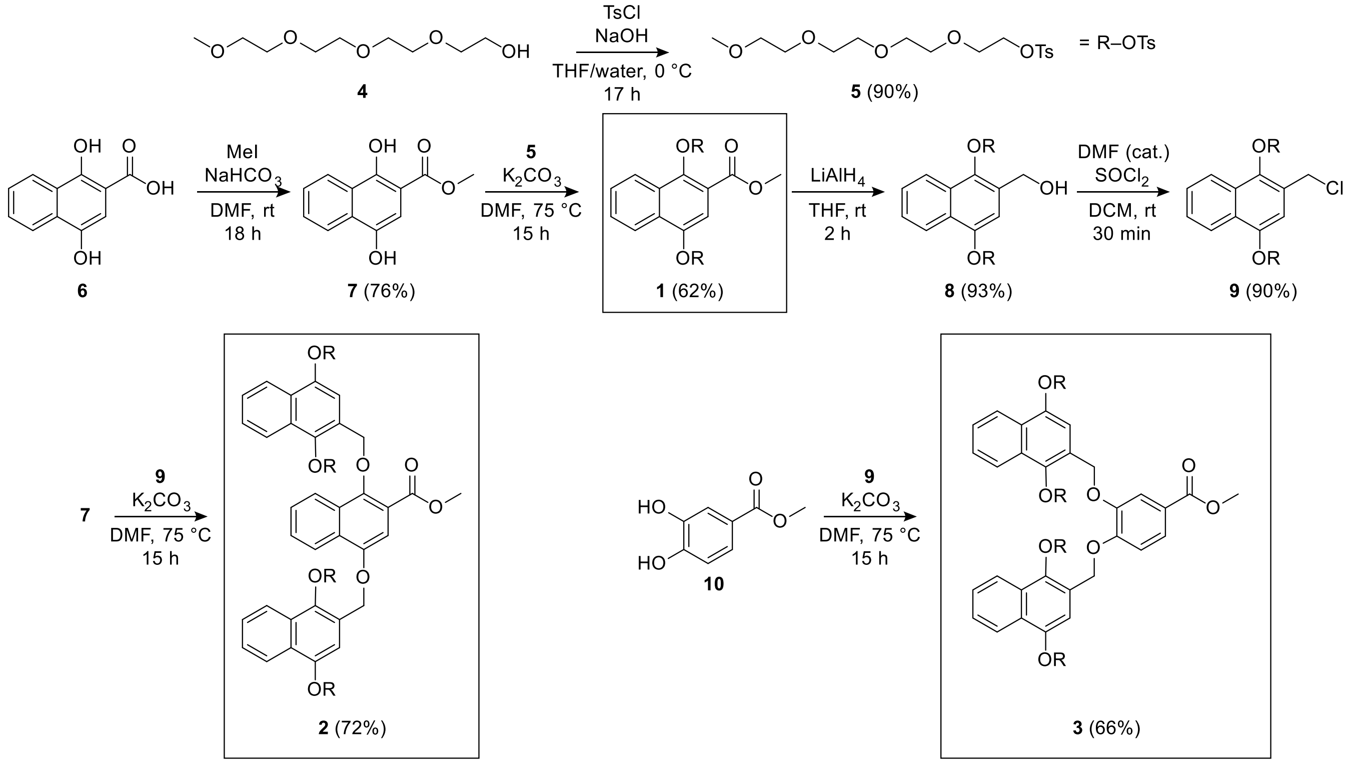

2,5,8,11-Tetraoxatridecan-13-yl 4-methylbenzenesulfonate (5).[1] Solid NaOH (5.76 g, 144 mmol, 1.5 equiv) and tetraethylene glycol monomethyl ether (**4**, 20.3 mL, 96.0 mmol, 1.0 equiv) were dissolved in a mixture of THF (280 mL) and water (40 mL). The solution was cooled to 0 °C. In a separate flask, TsCl (20.1 g, 106 mmol, 1.1 equiv) was dissolved in THF (50 mL) and the solution was added dropwise to the reaction flask. The solution was stirred at 0 °C for 17 h. The reaction mixture was poured into cold water (~5 °C, 100 mL) and then transferred to a separatory funnel. The aqueous layer was washed with CH_2Cl_2 (100 mL × 2). The combined organic layers were washed with HCl (1 M aq., 100 mL), dried with $MgSO_4$, and concentrated under vacuum to give **5** as a pale-yellow oil (31.3 g, 90%).

^{1}H NMR (500 MHz, DMSO-d_6) δ 8.61–8.50 (m, 2H), 8.23 (dt, J = 8.5, 2.1 Hz, 2H), 4.91–4.82 (m, 2H), 4.39–4.29 (m, 3H), 4.28–4.14 (m, 13H), 3.97 (s, 3H), 3.17 (s, 3H).

^{13}C NMR (126 MHz, DMSO-d_6) δ 146.08, 133.63, 131.32, 128.82, 72.47, 71.17, 70.99, 70.95, 70.90, 70.85, 70.77, 69.08, 59.23, 41.23, 41.06, 40.89, 40.73, 40.56, 40.39, 40.23, 22.27.

HRMS ESI m/z [M+Na]$^+$, calcd for $C_{16}H_{26}O_7Na^+$ 385.1291; found 385.1265.

Methyl 1,4-dihydroxy-2-naphthoate (7).[2] To a mixture of 1,4-dihydroxy-2-naphthoic acid (**6**, 5.00 g, 24.5 mmol, 1 equiv) and $NaHCO_3$ (2.26 g, 26.94 mmol, 1.1 equiv) in DMF (75 mL) was added dropwise MeI (1.8 mL,

29.39 mmol, 1.2 equiv). The reaction mixture was stirred at room temperature under N_2 for 18 h. NaCl (sat. aq., 25 mL) was added to the flask and the mixture was stirred vigorously for 10 min. The reaction mixture was transferred to a separatory funnel and HCl (1 M aq., 23 mL) was added. The aqueous layer was washed with diethyl ether (75 mL × 3). The combined organic layers were washed with LiCl (0.5 M aq., 100 mL × 4) and NaCl (sat. aq., 100 mL), dried with $MgSO_4$, and concentrated under vacuum. The product was purified by column chromatography (hexanes to 4:1 hexanes/EtOAc) to give **7** as a golden-brown solid (4.04 g, 76%).

^{1}H NMR (500 MHz, DMSO-d_6) δ 12.13 (s, 1H), 10.67 (s, 1H), 9.05 (dt, J = 8.1, 1.0 Hz, 1H), 8.92 (dt, J = 8.3, 1.0 Hz, 1H), 8.47 (ddd, J = 8.3, 6.9, 1.3 Hz, 1H), 8.40 (ddd, J = 8.2, 6.9, 1.3 Hz, 1H), 7.90 (s, 1H), 4.74 (s, 3H).

^{13}C NMR (126 MHz, DMSO-d_6) δ 171.72, 153.87, 146.33, 130.22, 129.93, 127.63, 126.04, 124.40, 123.37, 105.94, 104.96, 53.78.

HRMS ESI m/z [M–H]$^-$, calcd for $C_{12}H_9O_4^-$ 217.0506; found 217.0490.

Methyl 1,4-bis((2,5,8,11-tetraoxatridecan-13-yl)oxy)-2-naphthoate (1).[3] To a mixture of **7** (3.00 g, 13.8 mmol, 1 equiv) and K_2CO_3 (11.4 g, 82.5 mmol, 6.0 equiv) in DMF (70 mL) was added dropwise **5** (10.2 g, 28.2 mmol, 2.05 equiv). The reaction mixture was stirred at 75 °C under N_2 for 15 h. The flask was allowed to reach room temperature whereupon it was poured into water (210 mL). The mixture was transferred to a separatory funnel and the aqueous layer was washed with EtOAc (150 mL × 4). The combined organic layers were washed with LiCl (0.5 M aq., 225 mL × 3), water (100 mL × 2), and NaCl (sat. aq., 150 mL), dried with $MgSO_4$, and concentrated under vacuum. The product was purified by column chromatography (EtOAc to 9:1 EtOAc/MeOH) to give **1** as a red-orange oil (5.13 g, 62%).

^{1}H NMR (500 MHz, DMSO-d_6) δ 8.38–8.31 (m, 1H), 8.22–8.16 (m, 1H), 7.71–7.62 (m, 2H), 7.14 (s, 1H), 4.31–4.24 (m, 2H), 4.16–4.10 (m, 2H), 3.91–3.85 (m, 5H), 3.82–3.76 (m, 2H), 3.69–3.64 (m, 2H), 3.64–3.60 (m, 2H), 3.60–3.54 (m, 7H), 3.54–3.45 (m, 11H), 3.43–3.36 (m, 4H), 3.23–3.19 (m, 6H).

^{13}C NMR (126 MHz, DMSO-d_6) δ 166.10, 149.94, 149.56, 128.88, 127.98, 127.32, 123.57, 121.83, 119.08, 104.53, 74.80, 71.27, 71.25, 70.09, 69.96, 69.90, 69.88, 69.84, 69.81, 69.77, 69.71, 69.59, 69.56, 68.90, 68.11, 58.02, 52.20.

HRMS ESI m/z [M+Na]$^+$, calcd for $C_{30}H_{46}O_{12}Na^+$ 621.2882; found 621.2850.

1,4-Bis((2,5,8,11-tetraoxatridecan-13-yl)oxy)naphthalen-2-yl)methanol (8).[4,5] A solution of **1** (2.00 g, 3.34 mmol, 1 equiv) in THF (60 mL) was cooled to 0 °C whereupon $LiAlH_4$ (2.4 M in THF, 2.09 mL, 5.01 mmol, 1.5 equiv) was added dropwise. The reaction mixture was allowed to come to room temperature and was stirred for 2 h. A mixture of Na_2SO_4 and Celite (1:1 v/v) was added with stirring until the mixture ceased stirring and then resumed stirring, after which it was stirred vigorously for an additional 15 min. The mixture was vacuum filtered and the filtrate was concentrated under vacuum to give **8** as a pale-orange oil (1.77 g, 93%)

^{1}H NMR (500 MHz, DMSO-d_6) δ 8.12 (tdd, *J* = 9.0, 1.3, 0.7 Hz, 2H), 7.54 (ddd, *J* = 8.2, 6.8, 1.4 Hz, 1H), 7.49 (ddd, *J* = 8.2, 6.8, 1.3 Hz, 1H), 7.03 (s, 1H), 5.18 (t, *J* = 5.7 Hz, 1H), 4.70 (d, *J* = 5.7 Hz, 2H), 4.28–4.21 (m, 2H), 4.05–3.98 (m, 2H), 3.92–3.85 (m, 2H), 3.82–3.73 (m, 2H), 3.69–3.60 (m, 7H), 3.59–3.55 (m, 5H), 3.55–3.47 (m, 12H), 3.44–3.38 (m, 5H), 3.22 (s, 3H), 3.21 (s, 3H).
^{13}C NMR (126 MHz, DMSO-d_6) δ 150.28, 144.28, 130.59, 128.24, 126.46, 125.13, 121.96, 121.82, 105.44, 73.87, 71.25, 70.10, 70.02, 69.92, 69.90, 69.82, 69.78, 69.73, 69.59, 69.56, 68.99, 67.89, 58.01, 57.71.
HRMS ESI *m/z* $[M+Na]^+$, calcd for $C_{29}H_{46}O_{11}Na^+$ 593.2932; found 593.2920.

13,13'-((2-(Chloromethyl)naphthalene-1,4-diyl)bis(oxy))bis(2,5,8,11-tetraoxatridecane) (9).[5] A solution of **8** (1.77 g, 3.10 mmol, 1 equiv) in CH_2Cl_2 (75 mL) was cooled to 0 °C whereupon $SOCl_2$ (2.0 M in CH_2Cl_2, 1.9 mL, 3.72 mmol, 1.2 equiv) was added dropwise. The reaction mixture was allowed to come to room temperature and was stirred for 30 min. Water (40 mL) was added and the mixture stirred vigorously for 10 min. The mixture was transferred to a separatory funnel and the aqueous layer was washed with CH_2Cl_2 (50 mL × 3). The combined organic layers were washed with water (40 mL × 2) and NaCl (sat. aq., 40 mL), dried with $MgSO_4$, and concentrated under vacuum to give **9** as a pale-orange oil (1.64 g, 90%) that was immediately in the next reaction without further purification.
^{1}H NMR (500 MHz, DMSO-d_6) δ 8.16 (dt, *J* = 7.9, 1.7 Hz, 2H), 7.59 (dddd, *J* = 20.2, 8.1, 6.8, 1.4 Hz, 2H), 6.98 (s, 1H), 4.96 (s, 2H), 4.29–4.24 (m, 2H), 4.14–4.09 (m, 2H), 3.91–3.87 (m, 2H), 3.84–3.79 (m, 2H), 3.69–3.64 (m, 4H), 3.64–3.60 (m, 2H), 3.60–3.46 (m, 15H), 3.43–3.37 (m, 4H), 3.21 (d, *J* = 7.6 Hz, 6H).
^{13}C NMR (126 MHz, DMSO-d_6) δ 150.58, 146.10, 128.10, 127.01, 126.30, 126.18, 125.95, 122.46, 122.00, 106.46, 74.56, 71.27, 71.24, 70.09, 70.05, 69.94, 69.91, 69.83, 69.77, 69.59, 69.55, 68.92, 68.07, 58.03, 58.00, 41.59.

Methyl 1,4-bis((1,4-bis((2,5,8,11-tetraoxatridecan-13-yl)oxy)naphthalen-2-yl)methoxy)-2-naphthoate (2).[3] To a mixture of **7** (0.10 g, 0.48 mmol, 1.0 equiv) and K_2CO_3 (0.41 g, 2.88 mmol, 6.0 equiv) in DMF (15 mL) was added dropwise **9** (0.58 g, 0.98 mmol, 2.05 equiv). The reaction mixture was stirred at 75 °C under N_2 for 15 h. The flask was allowed to reach room temperature and poured into water (45 mL). The reaction mixture was transferred to a separatory funnel and the aqueous layer was washed with EtOAc (20 mL × 5). The combined organic layers were washed with LiCl (0.5 M aq., 25 mL × 3), water (20 mL × 2), and NaCl (sat. aq., 20 mL), dried with $MgSO_4$, and concentrated under vacuum. The product was purified by column chromatography (EtOAc to 9:1 EtOAc/MeOH) to give **2** as a gold oil (0.44 g, 72%).
^{1}H NMR (500 MHz, DMSO-d_6) δ 8.27 (dt, *J* = 6.7, 3.4 Hz, 1H), 8.26–8.17 (m, 5H), 7.70–7.54 (m, 6H), 7.46 (s, 1H), 7.21 (s, 1H), 7.16 (s, 1H), 5.51 (s, 2H), 5.31 (s, 2H), 4.33–4.25 (m, 4H), 4.17 (t, *J* = 4.3 Hz, 2H), 4.05–4.00 (m, 2H), 3.91 (dd, *J* = 5.6, 3.6 Hz, 2H), 3.89 (s, 5H), 3.80–3.76 (m, 2H), 3.73–3.63 (m, 6H), 3.61–3.34 (m, 47H), 3.20–3.15 (m, 12H).

^{13}C NMR (126 MHz, DMSO-d_6) δ 166.21, 150.54, 150.41, 150.06, 149.57, 146.32, 145.79, 128.88, 128.25, 128.15, 127.99, 127.45, 126.88, 126.78, 126.04, 125.97, 125.62, 124.91, 123.43, 122.34, 122.00, 119.56, 106.15, 106.08, 104.97, 74.83, 74.55, 72.17, 71.23, 70.10, 69.96, 69.83, 69.76, 69.71, 69.55, 68.96, 68.03, 65.41, 57.98, 52.28.
HRMS ESI *m/z* $[M+2Na]^{2+}$, calcd for $C_{70}H_{98}O_{24}Na_2^{2+}$ 684.3116; found 684.3111.

Methyl 3,4-bis((1,4-bis((2,5,8,11-tetraoxatridecan-13-yl)oxy)naphthalen-2-yl)methoxy)benzoate (3).[3] To a mixture of methyl 3,4-dihydroxybenzoate (**10**, 0.23 g, 1.36 mmol, 1.0 equiv) and K_2CO_3 (1.14 g, 8.15 mmol, 6.0 equiv) in DMF (17.5 mL) was added dropwise **9** (1.64 g, 1.36 mmol, 2.05 equiv). The reaction mixture was stirred at 75 °C under N_2 for 18 h. The flask was allowed to reach room temperature and poured into water (60 mL). The reaction mixture was transferred to a separatory funnel and the aqueous layer was washed with EtOAc (45 mL × 5). The combined organic layers were washed with LiCl (0.5 M aq., 50 mL × 3), water (45 mL × 2), and NaCl (sat. aq., 45 mL), dried with $MgSO_4$, and concentrated under vacuum. The product was purified by column chromatography (EtOAc to 9:1 EtOAc/MeOH) to give **3** as a yellow-orange oil (1.20 g, 66%).
^{1}H NMR (500 MHz, DMSO-d_6) δ 8.17–8.11 (m, 4H), 7.74 (d, *J* = 2.0 Hz, 1H), 7.67 (dd, *J* = 8.5, 2.0 Hz, 1H), 7.61–7.51 (m, 4H), 7.37 (d, *J* = 8.6 Hz, 1H), 7.00 (d, *J* = 8.5 Hz, 2H), 5.35 (d, *J* = 19.1 Hz, 4H), 4.14–4.04 (m, 4H), 4.02–3.91 (m, 4H), 3.84 (s, 3H), 3.72–3.36 (m, 56H), 3.19 (s, 12H).
^{13}C NMR (126 MHz, DMSO-d_6) δ 165.90, 152.69, 150.36, 150.28, 147.89, 146.13, 146.02, 128.15, 126.83, 126.77, 125.90, 125.09, 124.78, 123.65, 122.21, 121.96, 114.33, 113.04, 105.76, 74.68, 74.61, 71.23, 69.95, 69.90, 69.80, 69.74, 69.61, 69.55, 68.68, 67.71, 67.64, 65.70, 65.53, 57.98, 51.90.
HRMS ESI *m/z* $[M+2Na]^{2+}$, calcd for $C_{66}H_{96}O_{24}Na_2^{2+}$ 659.3038; found 659.3024.

2. Synthesis of A$\beta_{16–22}$ Peptide

Fragment 16–22 β amyloid peptide (A$\beta_{16–22}$) was a kind gift from the Nilsson group at the University of Rochester, synthesized through solid-phase peptide synthesis and purified using analytical high-pressure liquid chromatography (HPLC).[6] Lyophilized, purified peptide samples were dissolved in 60% MeCN in water with 1% trifluoroacetic acid and sonicated for 5 min. Peptide concentrations were analyzed via analytical HPLC using a reverse phase Phenomenex Gemini column (5 µm, C18, 110 Å, 250 × 4.6 mm, Phenomenex) on a Shimadzu LC-2010A for comparison to a concentration curve calibrated by amino acid analysis. The peptide solution was aliquoted into Eppendorf tubes, frozen, then lyophilized. Immediately prior to use, each lyophilized aliquot was dissolved in water to a final concentration of 500 µM.

3. UV-vis Spectroscopy

3.1. Materials and Methods

UV-vis spectra were recorded on an Agilent Cary 3500 Multicell Peltier. Spectra were recorded with a scanning speed of 600 nm/min, average time of 0.100 s, a data interval of 1 nm, and spectral bandwidth of 2.00 nm. Solutions were measured in a capped quartz cuvette (Starna Cells) with a path length of either 1 mm or 10 mm, selected to ensure the maximum absorbance was between 0.1 and 3. Solutions in 10 mm cuvettes were stirred with a magnetic stirrer bar at 600 rpm.

Solutions of **1** and **3** were prepared from aqueous stock solutions (1000 µM and 250 µM, respectively) by dilution to the appropriate concentration with water. Solutions of **2** were prepared from a stock solution (1000 µM) in MeCN (spectrophotometric grade, Thermo Scientific) by dilution to the appropriate concentration with water and MeCN.

For full-spectrum studies, solutions were heated at a rate of 0.5 °C/min from 20 to 75 °C, held at 75 °C for 10 min, then cooled from 75 to 20 °C. Spectra were recorded from 700 to 200 nm at 20, 40, 60 and 75 °C upon both heating and cooling.

For discrete wavelength studies, solutions were heated at a rate of 0.5 °C/min from 20 to 75 °C, held at 75 °C for 10 min, then cooled from 75 to 20 °C. This heat-hold-cool cycle was repeated three times. The absorbance at 217, 249, 295, and 343 nm was recorded at 1 °C intervals. Full-wavelength spectra from 700 to 200 nm were recorded at 20 °C at the start and end of the experiment (i.e., before the first heating and after the third cooling).

3.2. Additional UV Data

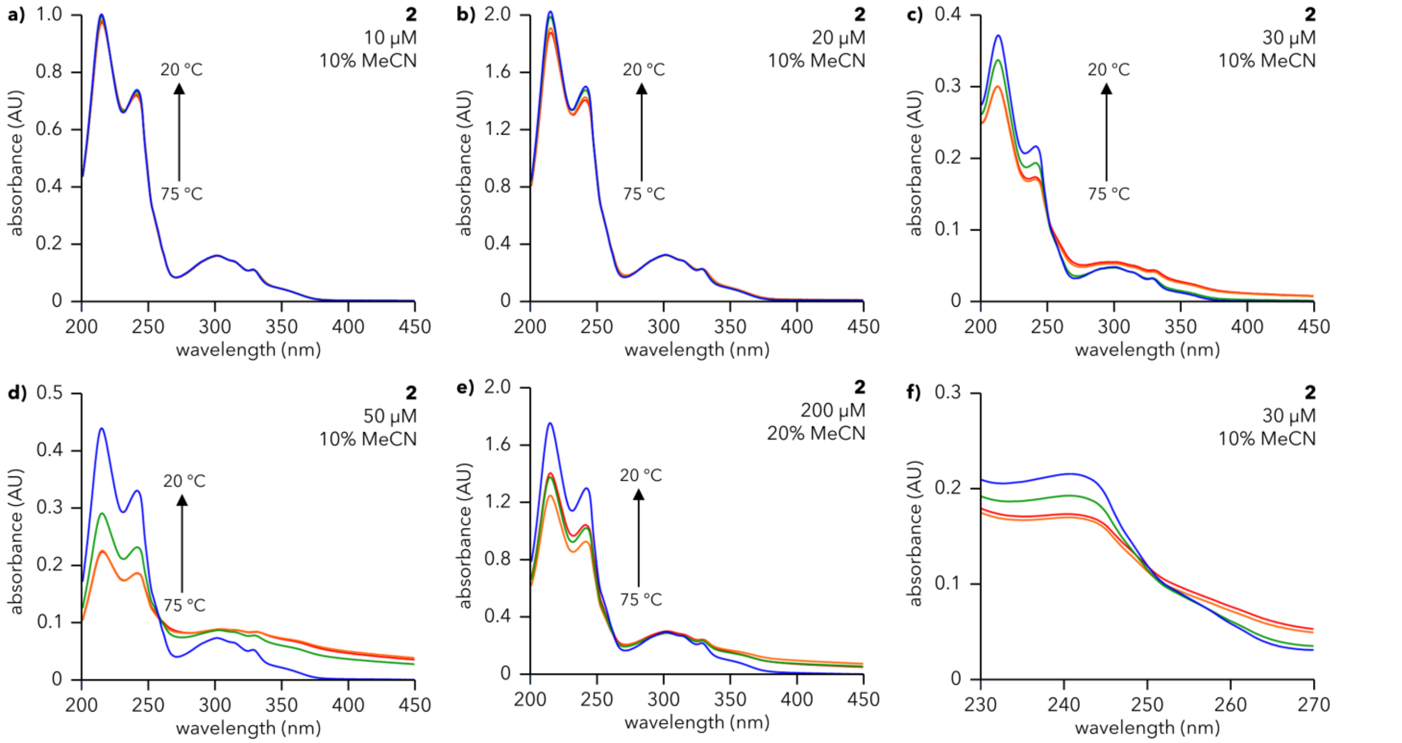

Figure S1. Assembly of 2 in aqueous MeCN. UV-vis spectra of **2** collected upon cooling solutions from 75 °C (red line) to 20 °C (blue line) at the indicated concentrations in (**a**–**d**) 10% MeCN in water and (**e**) 20% MeCN in water. Spectra were collected at 75, 60, 40, and 20 °C. Spectra in **a** and **b** collected in a cuvette with path length (*l*) of 10 mm; spectra in **c**–**e** were collected in a cuvette with *l* = 1 mm. (**f**) Magnified section of the spectrum in **c**.

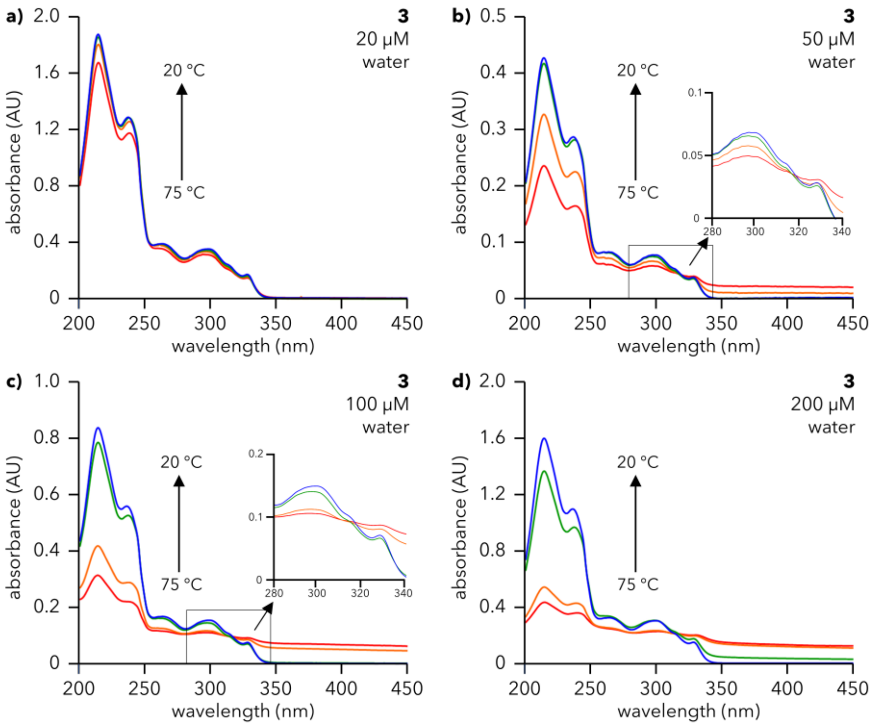

Figure S2. Assembly of 3 in water. UV-vis spectra of **3** collected upon cooling aqueous solutions at the indicated concentrations from 75 °C (red line) to 20 °C (blue line). Spectra were collected at 75, 60, 40, and 20 °C. Spectra in **a** collected in a cuvette with path length (l) of 10 mm; spectra in **b–d** were collected in a cuvette with l = 1 mm.

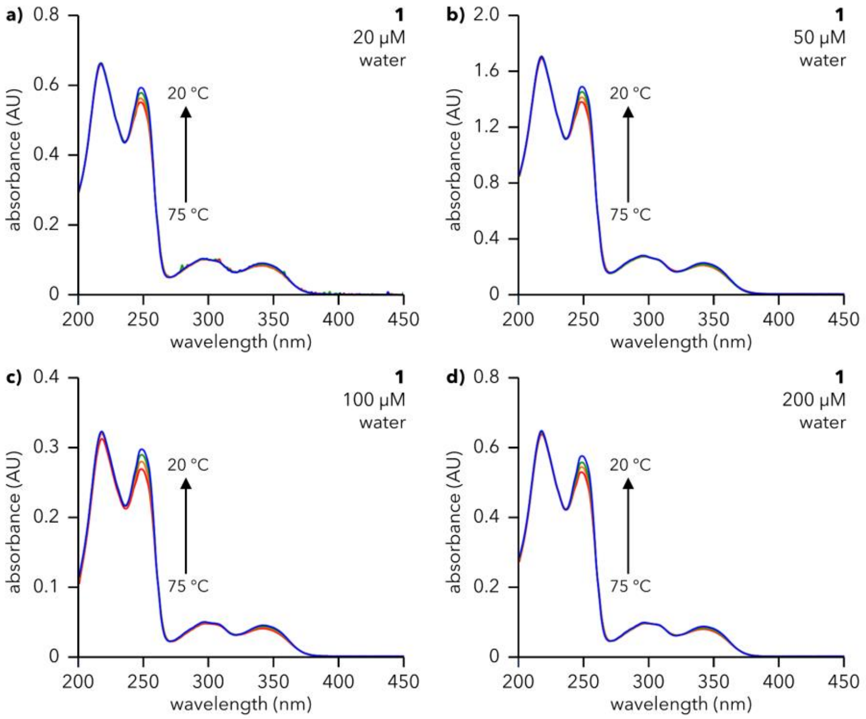

Figure S3. Assembly of 1 in water. UV-vis spectra of **1** collected upon cooling aqueous solutions at the indicated concentrations from 75 °C (red line) to 20 °C (blue line). Spectra were collected at 75, 60, 40, and 20 °C. Spectra in **a** and **b** collected in a cuvette with path length (l) of 10 mm; spectra in **c** and **d** were collected in a cuvette with l = 1 mm.

3.3. Reversibility Study

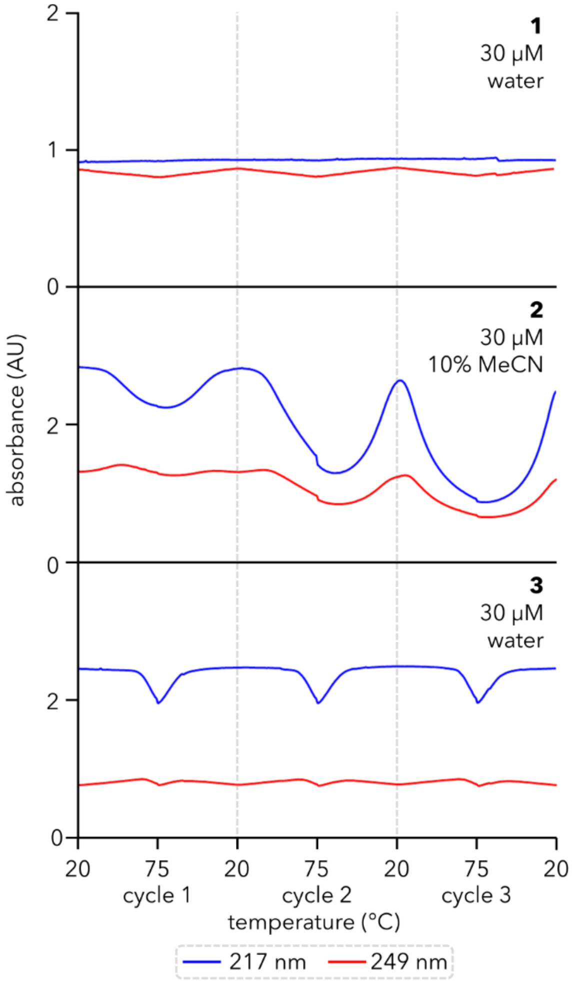

Figure S4. Multiple-cycle UV-vis of 1, 2, and 3 in solution. Absorbance at 217 nm (blue) and 249 nm (red) of **1** (30 µM in water), **2** (30 µM in 10% MeCN), and **3** (30 µM in water) collected every 1 °C during three heating and cooling cycles. In each cycle, solutions were heated to 75 °C, held at 75 °C for 10 min, and cooled to 20 °C. Heating and cooling rates: 0.5 °C/min. Spectra were collected in a cuvette with path length (*l*) of 10 mm.

4. Atomic Force Microscopy

4.1. Materials and Methods

Atomic force microscopy (AFM) measurements were performed at room temperature on an Asylum MFP-3D-BIO AFM (Oxford Instruments) using the Asylum Research version 12 software. AFM was operated in tapping mode with Olympus made AC240TS-R3 silicon cantilevers with spring constants of ≈ 2 N/m at their resonant frequencies of ≈ 70 kHz. The scan rate was 0.75 Hz per line, and the acquisition time for one image is ≈ 6 min. Image analysis was performed with Igor Pro 6.3.7.2.

Solutions of **1** and **3** were prepared from aqueous stock solutions (1000 µM and 250 µM, respectively) by dilution to the appropriate concentration with water. Where noted, samples were heated from 25 °C to 75 °C at a rate of 5.0 °C/min and subsequently cooled from 75 °C to 25 °C at a rate of 0.5 °C/min using an Agilent Cary 3500 Multicell Peltier.

Mica was used as the substrate for analysis for **1** and **3** because of its hydrophilic properties. Samples were prepared by spin-coating 50 µL of the solution at 3,000 rpm for 60 s onto grade V-4 freshly cleaved mica substrate (SPI Supplies).

Solutions of Aβ_{16-22} were prepared from a 500 µM aqueous stock solution and diluted to 200 µM with water.

Solutions containing Aβ_{16-22} and **1** or **3** were prepared from aqueous stock solutions of Aβ_{16-22} (500 µM), **1** (500 µM), and **3** (250 µM) by dilution to the appropriate concentration with water. Samples containing Aβ_{16-22} required the use of highly oriented pyrolytic graphite (HOPG, SPI Supplies) because the charged mica surface did not allow the peptide to lie flat for imaging. Samples were prepared by spin-coating 50 µL of the solution at 3,000 rpm for 60 s onto HOPG substrate.

4.2. Additional AFM Data

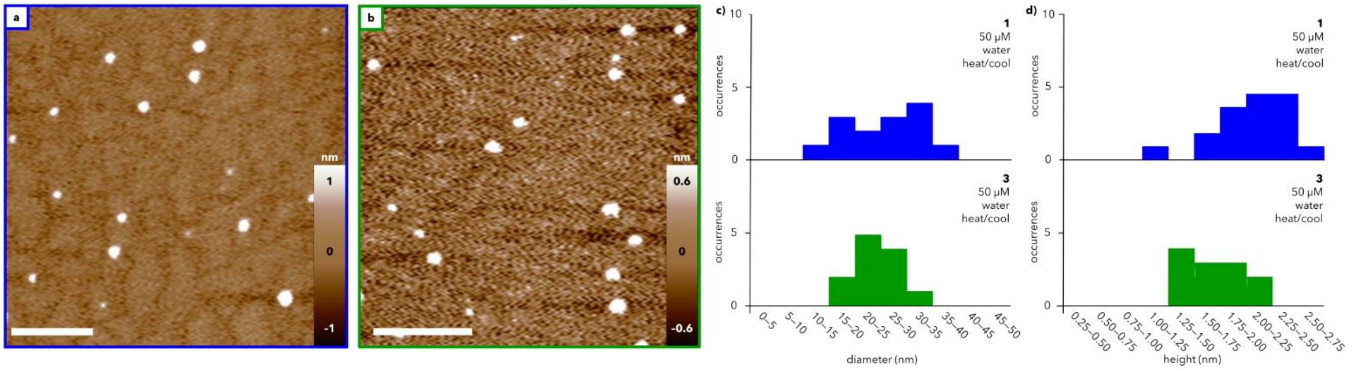

Figure S5. AFM of 1 and 3 at 50 µM. (**a**, **b**) AFM height images of (**a**) **1** and (**b**) **3** spin-coated on mica substrates prepared from 50 µM solutions in water that were heated to 75 °C and subsequently cooled to 25 °C (at 0.5 °C/min). Scale bars = 200 nm. Histograms showing the (**c**) diameters and (**d**) heights of particles in (**a**, **b**), denoted by color.

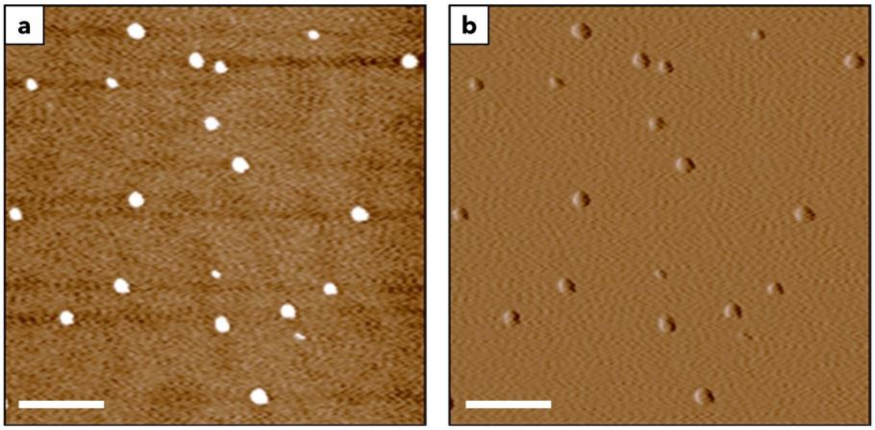

Figure S6. AFM of 1 at 30 µM. AFM (**a**) height and (**b**) amplitude images of **1** spin-coated on a mica substrate prepared from a 30 µM solution in water that was heated to 75 °C and subsequently cooled to 25 °C (at 0.5 °C/min). Scale bars = 200 nm. Note that (**a**) is identical to Figure 3a in the main text.

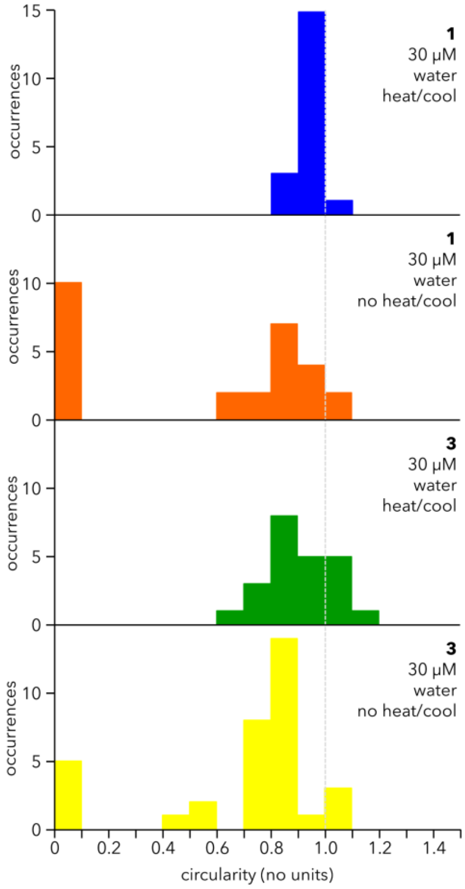

Figure S7. Degree of circularity of 1 and 3. Circularity is a measure of the ratio of the area of the particle divided by the area of a circle with an equivalent perimeter length. Area, perimeter, and circularity were determined from the AFM height data shown in Figure 3a–d. The more circular a particle, the closer to 1 this value will be. A circularity of 1 denotes a perfectly circular particle while a circularity of 0 denotes a highly irregular, non-circular particle. Broken grey line at circularity = 1 is a guide for the eye.

5. Transmission Electron Microscopy

5.1. Materials and Methods

Transmission electron microscopy (TEM) measurements were performed on a Hitachi 7650 TEM working at 80 kV with an attached 11-megapixel Gatan Erlangshen digital camera for image capture. Images were recorded using Digital Micrograph software.

Solutions of **1** and **3** were prepared in the same way as for AFM (SI Section 4.1). For staining, 10 µL of the sample solution together with 10 µL of phosphotungstic acid (PTA) solution (2% aq., pH 6.5) were loaded onto 200 mesh carbon/formvar copper grids and incubated for 2 min. The excess solution was wicked off with filter paper and the grids were allowed to dry.

5.2. TEM Data

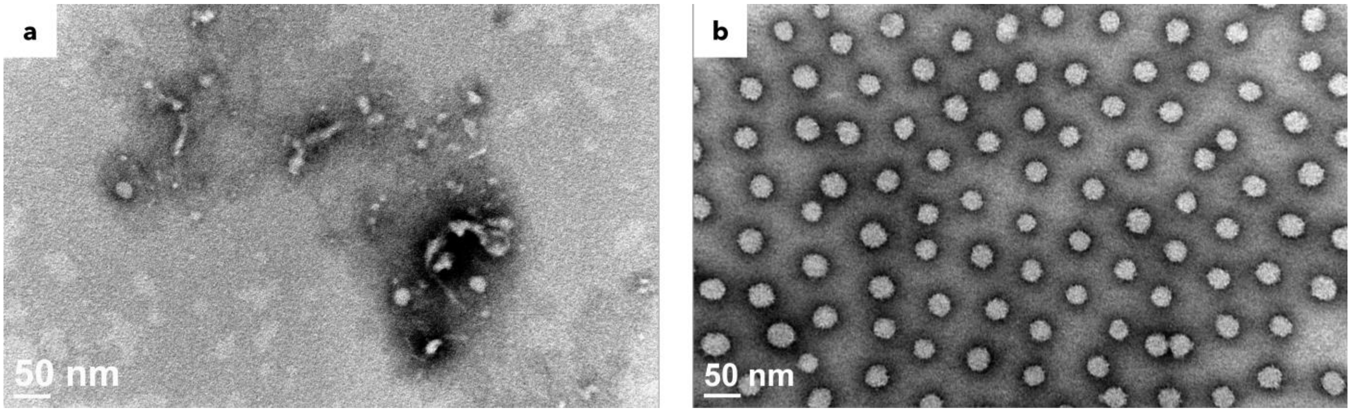

Figure S8. TEM of dendrons at 30 µM. TEM images of (**a**) **1** and (**b**) **3** after negative staining with phosphotungstic acid.

TEM images of **1** (Figure S8a) show poorly assembled morphologies with only a few particle-like structures. Given the amphilphilic nature of dendrons **1** and **3** and the significantly smaller hydrophobic region in **1** compared to **3**, we hypothesize that the PTA stain may be disrupting the assembly of **1**.

6. Fluorescence Spectroscopy

6.1. Materials and Methods

Fluorescence emission spectra were recorded at room temperature on a Spex Fluoromax-3 fluorometer (Jobin-Yvon Horiba) from 400 to 700 nm, with an excitation wavelength of 488 nm, slit widths of 2 nm, and integration time of 0.5 s. Solutions were measured in a capped quartz fluorescence cuvette (Starna Cells) with a path length of 10 mm.

Solutions containing rhodamine 6G (Thermo Fisher Scientific) and **1** or **3** were prepared from aqueous stock solutions of rhodamine 6G (100 µM), **1** (1000 µM), and **3** (250 µM) by dilution to the appropriate concentration with water.

6.2. Fluorescence Studies with Rhodamine 6G

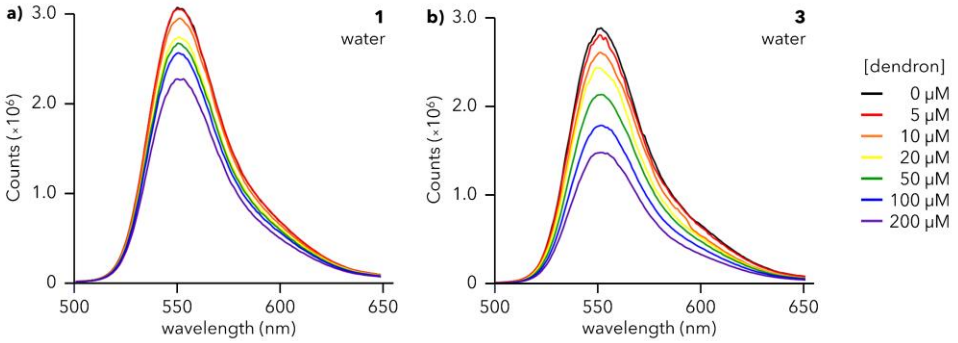

Figure S9. Fluorescence emission of rhodamine 6G with 1 or 3. Emission spectra of solutions of rhodamine 6G (1 µM) with various concentrations of (**a**) **1** or (**b**) **3** in water. Excitation wavelength = 488 nm. Spectra were recorded at ambient temperature (~23 °C).

7. Circular Dichroism Spectroscopy

7.1. Materials and Methods

Circular dichroism (CD) spectra were recorded at 25 °C on a Jasco J-1100 circular dichroism spectrophotometer from 190 to 260 nm with a scanning speed of 50 nm/min, data integration time of 4 s, and bandwidth of 1 nm. Solutions were measured in a capped quartz cuvette (Starna Cells) with a path length of either 1 mm or 10 mm, selected to ensure the maximum HT was less than 800 V.

Solutions of **1** and **3** were prepared from aqueous stock solutions (1000 µM and 250 µM, respectively) by dilution to the appropriate concentration with water.

Solutions of A$\beta_{16–22}$ were prepared from a 1000 µM aqueous stock solution and diluted to 200 µM with water.

Solutions containing A$\beta_{16–22}$ and **1** or **3** were prepared from aqueous stock solutions of A$\beta_{16–22}$ (1000 µM), **1** (500 µM), and **3** (250 µM) by dilution to the appropriate concentration with water.

7.2. Additional CD Spectra

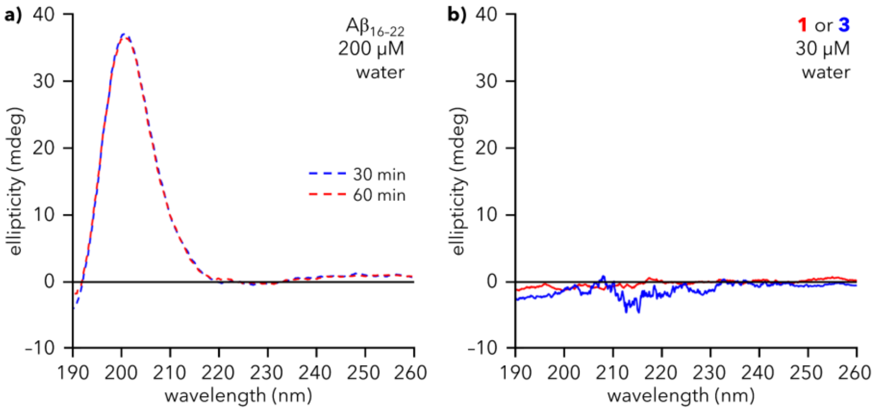

Figure S10. CD spectra of individual components in solution. (**a**) CD spectra of A$\beta_{16–22}$ in water (200 µM) collected 30 min (blue) and 60 min (red) after dissolution. (**b**) CD spectra of **1** (red) or **3** (blue) in water (30 µM). All spectra were collected at 25 °C. The spectrum of **1** was collected in a cuvette with path length (*l*) of 10 mm; the spectra of **3** and A$\beta_{16–22}$ were collected in a cuvette with *l* = 1 mm.

8. Amyloid Binding Assays

8.1. Thioflavin T

Fluorescence emission spectra were recorded at room temperature on a Spex Fluoromax-3 fluorometer (Jobin-Yvon Horiba) from 300 to 650 nm, with an excitation wavelength of 350 nm, slit widths of 2 nm, and integration time of 0.5 s. Solutions were measured in a capped quartz fluorescence cuvette (Starna Cells) with a path length of 10 mm.

Solutions of thioflavin T (ThT, Thermo Fisher Scientific) and mixtures of ThT with **3** were prepared from aqueous stock solutions (500 µM and 100 µM, respectively) by dilution to the appropriate concentration with water. Spectra were recorded at room temperature.

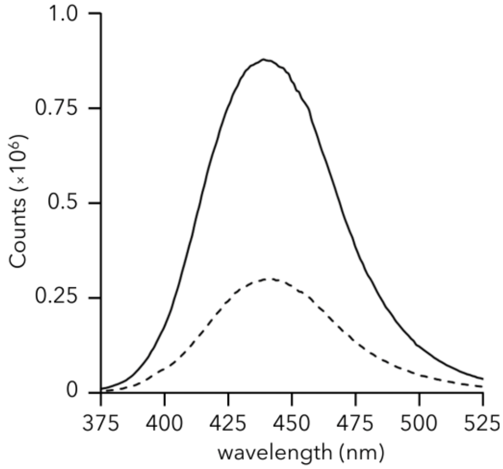

Figure S11. Thioflavin T fluorescence binding assay. Emission spectra of ThT (15 µM, broken line) and ThT (15 µM) with **3** (30 µM, solid line) in water. Excitation wavelength = 350 nm. Spectra were recorded at ambient temperature (~23 °C). The increase in the fluorescence of ThT in the presence of **3**, indicative of an interaction between ThT and **3**, prevents the fluorescent turn-on of ThT being used as an indicator of amyloid fibrillation.

8.2. Congo Red

For Congo red (CR) binding assays, solutions of CR (Sigma-Aldrich), **1**, or **3** were prepared from aqueous stock solutions (2000 µM, 1000 µM, and 250 µM, respectively) by dilution to the appropriate concentration with water. Solutions of $A\beta_{16\text{–}22}$ were prepared from aqueous stock solutions (1000 µM) by dilution to the appropriate concentrations with water. Solution mixtures were prepared from their respective stock solution and diluted to the appropriate concentration with water. Spectra were recorded between 200 and 800 nm as soon as practicable after sample preparation (0 h, Figure S12, broken lines) and 60 minutes after the first spectrum was recorded (1 h, Figure S12, solid lines), using the instrument parameters described in SI Section 3.1.

After spectra were collected at the 1 h timepoint, the cuvettes were stored at 25 °C in the dark for 25 h. The cuvettes were mixed by inverting them several times, and their UV spectra were recorded (Figure S13a).

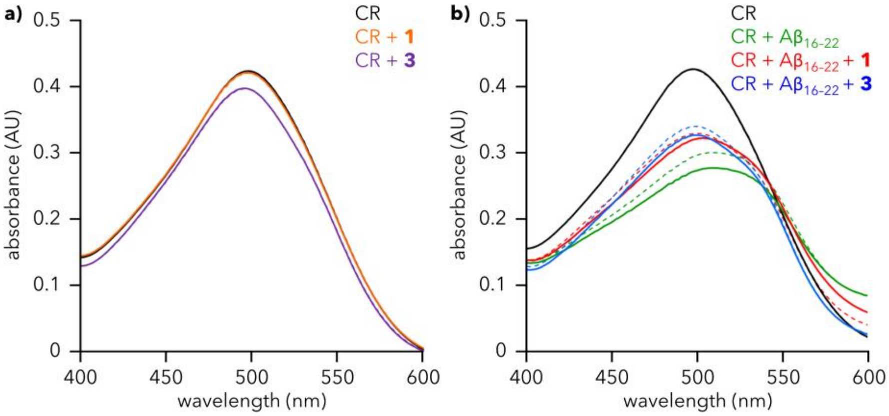

Figure S12. Congo red (CR) UV absorbance binding assay. UV-vis spectra of **(a)** CR (100 µM) and CR (100 µM) with **1** (30 µM) or **3** (30 µM) and **(b)** CR (100 µM), CR (100 µM) with $A\beta_{16–22}$ (200 µM), and CR (100 µM) with $A\beta_{16–22}$ (200 µM) and **1** (30 µM) or **3** (30 µM) at 0 h (broken lines) and 1 h (solid lines). Spectra in **a** and **b** were collected in a cuvette with path length (*l*) of 1 mm at 25 °C.

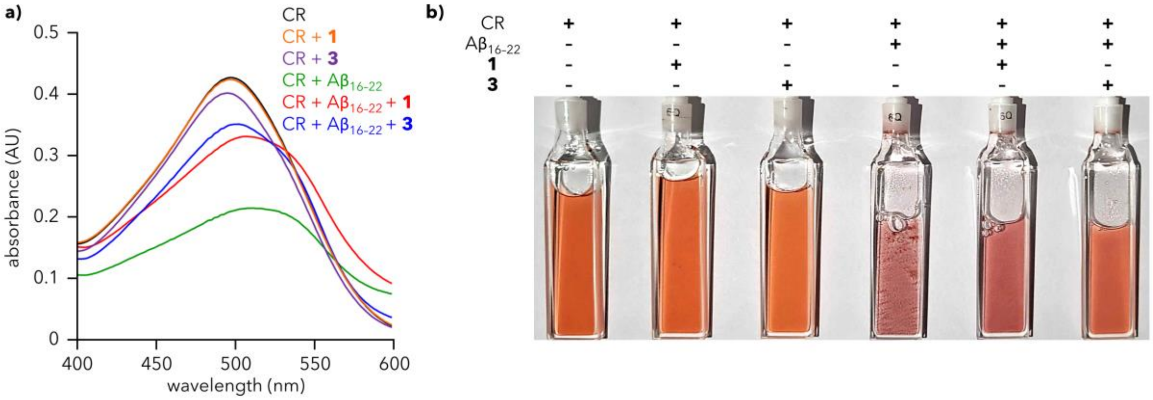

Figure S13. Samples of CR and $A\beta_{16–22}$ after 26 h. (a) UV-vis spectra of the samples from Figure S12 after incubation at 25 °C for 26 h after sample preparation. Spectra were collected in a cuvette with path length (*l*) of 1 mm at 25 °C. **(b)** Photograph of the samples from **a** immediately after UV spectra were collected. Formation of a precipitate and a change in color are visible in cuvettes containing CR + $A\beta_{16–22}$ and CR + $A\beta_{16–22}$ + **1**.

9. References for the Supporting Information

1. J. Li, M. Chen, S. Zhou, H. Li and J. Hao, Self-assembly of fullerene C60-based amphiphiles in solutions, *Chem. Soc. Rev.*, 2022, **51**, 3226–3242.

2. P. C. Bulman Page, Y. Chan, A. H. Noor Armylisas and M. Alahmdi, Asymmetric epoxidation of chromenes mediated by iminium salts: Synthesis of mollugin and (3S,4R)-trans-3,4-dihydroxy-3,4-dihydromollugin, *Tetrahedron*, 2016, **72**, 8406–8416.

3. C. Roche, H.-J. Sun, P. Leowanawat, F. Araoka, B. E. Partridge, M. Peterca, D. A. Wilson, M. E. Prendergast, P. A. Heiney, R. Graf, H. W. Spiess, X. Zeng, G. Ungar and V. Percec, A supramolecular helix that disregards chirality, *Nat. Chem.*, 2016, **8**, 80–89.

4. V. Percec, C.-H. Ahn, W.-D. Cho, A. M. Jamieson, J. Kim, T. Leman, M. Schmidt, M. Gerle, M. Möller, S. A. Prokhorova, S. S. Sheiko, S. Z. D. Cheng, A. Zhang, G. Ungar and D. J. P. Yeardley, Visualizable Cylindrical Macromolecules with Controlled Stiffness from Backbones Containing Libraries of Self-Assembling Dendritic Side Groups, *J. Am. Chem. Soc.*, 1998, **120**, 8619–8631.

5. B. E. Partridge, L. Wang, D. Sahoo, J. T. Olsen, P. Leowanawat, C. Roche, H. Ferreira, K. J. Reilly, X. Zeng, G. Ungar, P. A. Heiney, R. Graf, H. W. Spiess and V. Percec, Sequence-Defined Dendrons Dictate Supramolecular Cogwheel Assembly of Dendronized Perylene Bisimides, *J. Am. Chem. Soc.*, 2019, **141**, 15761–15766.

6. C. W. Jones, C. G. Morales, S. L. Eltiste, F. E. Yanchik-Slade, N. R. Lee and B. L. Nilsson, Capacity for increased surface area in the hydrophobic core of β-sheet peptide bilayer nanoribbons, *J. Pept. Sci.*, 2021, **27**, e3334.
